# Expectations of new technologies in nursing care among hospital patients in Germany – an interview study

**DOI:** 10.3389/fpsyg.2023.1227852

**Published:** 2023-09-14

**Authors:** Ronny Klawunn, Urs-Vito Albrecht, Marie-Luise Dierks

**Affiliations:** ^1^Department for Patient Orientation and Health Education, Institute for Epidemiology, Social Medicine and Health Systems Research, Hannover Medical School (MHH), Hanover, Germany; ^2^Research Group Digital Medicine, Medical School EWL, Bielefeld University, Bielefeld, Germany

**Keywords:** patient perspective, nursing care, implementation, technical innovations, expectations, evaluative qualitative content analysis, assistive technology, Germany

## Abstract

**Introduction:**

New technologies will be increasingly available for nursing care, including robots, patient mobilisation devices, digital event detection or prevention equipment. Technologies are expected to support nurses, increase patients’ safety and reduce costs. Yet, although these technologies will significantly shape patients’ experience, we need to learn more about patients’ perspectives regarding new technology in care. This study aims to investigate attitudes, expectations, worries and anticipated implementation effects of new assistive technology in nursing care by patients.

**Methods:**

Qualitative, guided, semi-open interviews were conducted. The recruitment was carried out in a trauma surgery ward of a university hospital in Germany. Eight different technologies were presented via video clips and additional information to the patients, followed by in-depth discussions. The interviews were analysed using qualitative evaluative content analysis. The Consolidated Criteria for Reporting Qualitative Research (COREQ) Checklist was used to ensure study quality.

**Results:**

Study participants anticipate different outcomes for the implementation of new nursing technology: (1) For patients, they consider the potential for improvement in health and well-being as well as for their hospital stay experience, but also fear possible health risks or social or emotional factors like loss of autonomy or loneliness. (2) For professional nurses, participants expect relief from physically stressful work routines; however, they might be replaced by machines and lose their employment (3) For the nursing process, safety and quality improvements for care delivery may encounter a negative quantification of human life and risks of constant surveillance.

**Conclusion:**

Patients identify opportunities, challenges and shortcomings of nursing technology implementation. They describe nuanced and mixed accounts of patients’ perspectives that are structured in a ‘continuum of anticipated effects’ of implementing technology in our article. The results can inform future implementation strategies.

## 1. Background

Novel assistive technologies like (emotional, service or care) robots, incidence detection, mobilisation devices, or digital communication have become more available for nursing care settings. Such technologies will be used more widely in the future to meet the needs of people in care and to support the work of caregivers or mitigate workforce shortages (World Health Organisation, 2015). Technology is often seen as a valuable and inevitable development that can help to complement nursing processes (Locsin, 2017). The World Health Organisation sees digital technologies as a way to reduce inefficiencies in healthcare and sees the expansion of digital infrastructure and digital competencies as a method for strengthening the health workforce (World Health Organisation, 2022).

Whether new technologies will indeed fulfil the expectations placed on them in terms of a sociotechnical innovation (i.e., the change of social contexts using technical devices) in the various sectors of nursing care (acute inpatient care, long-term care, outpatient care) is currently open for debate (Hülsken-Giesler et al., 2022). However, the accelerated uptake of technology already raises concerns and fears of negative uncontrolled and unintended consequences. For example, while care is essentially a physical and social encounter between caregiver and caretaker, there might be an increase of care for devices to the disadvantage of care for people [see the debate on high-tech vs. high-touch in care (Waidley, 2019)]. Another problem could arise from technologies that simulate social relations toward the caretaker [for the case of emotional robots, see (Lipp and Maasen, 2019)], which raises questions about whether robots can provide human care at all (Coghlan, 2021). Therefore, new technology in nursing raises the question of how technology can be used in nursing care in a supportive, but not replacing manner.

Although numerous problems can be identified, some strategies for sound technology implementation practice can also be formulated: training of digital skills (Kaihlanen et al., 2021), the creation of a positive attitude among health professionals toward technology (Nadav et al., 2021) as well as an extensive introduction for professionals to technology operation (Albrecht et al., 2013) could be mentioned for this. Looking at the German healthcare system (in which this study is also situated), surveys find open, interested and curious attitudes toward new technologies among professional nurses. For example, one study showed that nurses tend to have an open attitude toward assistive technology and generally see it as useful and user-friendly (Merda et al., 2017). In contrast, the respondents were rather ambivalent about the use of robotics, as negative expectations of its use were pronounced more frequently (*ibid.*). Another study explored nurses’ expectations with regard to 10 products from the field of innovative assistive technologies. It was found that all technologies trigger positive expectations with regard to physical relief, documentation activities and patient monitoring (Zentrum für Qualität in der Pflege, 2019). Solely in the case of social and emotional assistance for patients, respondents are not clear about whether new technologies may have a positive effect (*ibid.*).

Accordingly, much is known about healthcare workers’ perspectives and implementation strategies to facilitate technology transition into practice. However, as new assistive technology affects patients as well, their perspectives also have to come into focus (Archibald and Barnard, 2018).

### 1.1. The importance of the patient’s perspective in emerging nursing technology

When it comes to new technology, patients may have different positions: they are vulnerable as a patient, seeking care for their health problems and enduring accompanying conditions, such as pain and psychological distress while negotiating through the healthcare institution (Fassin, 2008). At the same time, they become users or beneficiaries of new technology, which may result in new demands and skills like how to set up technology or which data one wants to share – worries about data security for patients might be relevant, as was examined by Illiger (Illiger et al., 2014).

Parallel to the expectation that patients make informed decisions and thus have a choice in the course of their treatment, the role of the technology user, who is both competent in the use of technology and able to deal confidently with its possibilities and limitations, is becoming increasingly relevant. In this regard, the use of technology in the care relationship represents more than the mere application of a tool. Instead, it is intertwined with expectations and practices of health care, thereby involving different actors like professional nurses and patients alike (Mol, 2008).

For patients, especially for those with chronic conditions, it is central to treat not only the physical health, because the management of the disease or its recovery depends on emotional factors that can, for instance, influence the experience of pain, the social integration into the society or the quality of life (Male et al., 2016). Technology can provide methods to support individualised, tailored health interventions and promote coping, emotional management, vitality, and disease acceptance (Durosini et al., 2022). For instance, this could take the form of robots that increase a person’s autonomy or keep patients company (Wright, 2023). Technologies like AI-based systems could also help to visualise treatment-relevant behaviour or body parameters for healthcare professionals, which in turn could enable interventions to be better adapted to patients.

However, there is also a risk, as technology has a standardising tendency (Bächle, 2019). It is not clear, for example, whether technology will individualise care or whether it will foster standardised care interventions. It is open whether technology is able to provide an individual response to patient needs or if the emotional state of a patient requires personal contact that can only be realised by a human being. To better understand what patients expect from a well-designed technology, how it can be used in an individualised care context – for instance to increase autonomy or health conditions – and which barriers and risks they anticipate, more is needed to know about how patients perceive technology implementation. For this reason, an interview study was conducted to investigate:

*How do patients perceive the emergence of novel forms of technology into nursing care, and what effects do patients anticipate for technology implementation processes?*

### 1.2. Study context

The interviews are part of the ‘Centre of Implementing Nursing Care Innovations’ (funding by the German Federal Ministry of Education and Research, funding number 16SV7892K). The project aims to identify nursing technology available on the market, select it with professional nurses’ participation and implement it in a trauma surgery ward of a university hospital in the north of Germany (Hechtel et al., 2021; Klawunn et al., 2021). After implementation, it will be examined how new assistive devices work in the everyday practice of nursing professionals, whether technology supports nurses and patients and helps to improve working processes. Even if the implementation activities in the research project are primarily designed together with nursing professionals, it is also the dedicated goal of the project to explore the perspective of patients and to consider this perspective in the subsequent implementation of new technologies. For this reason, this study is a component of the larger research project and stands alongside other activities that are reported elsewhere.

## 2. Methods

### 2.1. Study design

To openly explore the perspectives and attitudes of patients, a qualitative research approach was used with semi-structured, guideline-based in-depth interviews (Green and Thorogood, 2004b). To encourage the interviewees to reflect on new technologies, examples of technical devices for care were demonstrated with concrete examples. With stimulus based on texts and video clips, study participants were asked to share their ideas of technology implementation effects embedded within personal and culturally based values on health care, nursing and technology (Törrönen, 2002) (a description of sampling and recruitment strategy can be found in subsection 2.2).

The research team selected eight technology examples. They should be (1) innovative products so that the likelihood that patients have already heard of the technologies should be low. (2) The selected products should reflect the range of currently available product types or properties and task profiles from the field of innovative care technologies. The products should differ from each other so that a broad range of products and diverse responses are possible. (3) The technology examples should already be in preparation for implementation in the study ward or at least potentially applicable to the hospital setting. The eight technologies are described in Table 1.

**Table 1:** Description of the presented technology examples.

Name and short description of technology	Description*	Manufacturer and online resource
Tec 1 ‘Bedside Terminal’ *Bedside multimedia terminal*	The bedside system can be placed at the patient’s bedside and perform various tasks. Films, music and other multimedia offerings can be used. Patients’ individual needs can be communicated to the nursing staff (e.g., a request for coffee) or treatment plans can be accessed. The input takes place on touch screen.	Bewatec https://en.bewatec.com/
Tec 2 ‘Pflegebrille’ [Nursing glasses] *Augmented reality nursing glasses for practice guidance and vital sign documentation*	The nursing glasses are electronic glasses with an augmented reality function. They can be worn by nursing professionals or family members. Augmented reality here means that information on the glass becomes visible to the wearers. This information is intended to support caregivers in their activities. Since the care glasses are operated by head movement or by speech, the hands remain free and the instructions on the “screen” (the glasses) can be implemented directly.	Technical University Clausthal https://pflegebrille.de/index.php/de/pflegebrille
Tec 3 ‘Ekamove’ *Mattress add-on for re-positioning patient*	The Ekamove system is an electronically controlled inflatable chamber that is slid under a patient’s mattress. By inflating the chamber on one side at a time, the patient is turned alternately to the left or right side. This is intended to prevent the occurrence of pressure ulcers.	Ekamed https://www.eka.med.de/en/
Tec 4 ‘JustoCat’ *Emotional robot in the form of a house cat to calm dementia patients*	The JustoCat is an interactive robotic cat to activate the memory associated with real pets. In this way, it might have a calming effect on people with dementia. The robotic cat mimics many of the characteristics of a real cat and uses the memory that many people have of interacting with cats. Through sensors, it can respond to touch, such as petting or shaking, and respond with cat sounds or vibrations, which resemble a cat’s purr.	Robyn Robotics https://www.robicare.de/produkt/justocat/
Tec 5 ‘Lea’ *Tablet-equipped rollator to, among other features, support walking routes, set off fall alarms*	The Lea rollator is a computer-assisted mobility aid with many functions that can help people with daily routine activities. The rollator provides assistance in getting up and moving around safely - for example, it detects falls and can then automatically trigger an alarm. The rollator can be called by remote control and automatically approach the patient’s bed, for example. A built-in computer screen can be used, for example, to make telephone calls, schedule and view appointments.	Spark design and innovation, Robot Care Systems http://www.robotikworld.com/lea/
Tec 6 ‘Relay’ *Autonomous driving robot with access secured transport box*	The Relay service robot is a self-propelled transport robot developed for operation in a hospital. It can, for example, transport medications or laboratory samples (urine, blood, etc.) through the hospital’s corridors and elevators and drive them to a specific location. An access card secures access to the robot’s transport chamber so that the samples arrive safely at their destination.	Relay Robotics https://www.relayrobotics.com/
Tec 7 ‘Carbon Hand’ *Mobile exoskeleton for the hand to support gripping force*	The carbon hand is a robotic glove that is worn over a hand and supports the natural movements of the hand, such as grabbing and holding. By detecting the movement with the help of sensors in the glove, the wearer’s existing muscles can be artificially strengthened. Heavy loads on the wearer’s skeleton, muscles and tendons are reduced.	Exxomove https://www.exxomove.de/handmobilitaet
Tec 8 ‘Texible Wisbi’ *Incontinence and bed exit detection mat that issues an alert to a smartphone*	Textible Wisbi is a mat that is placed on the bed sheet. Through electronic sensors, the pad can detect urine. Thus, it is able to sound an alarm if a person with incontinence wets the bed unnoticed. It also detects when a person gets out of bed and can then sound an alarm. For example, persons who are not supposed to leave their bed can be protected by having the caregiver check on them immediately.	Texible https://www.texible.com/

* The brief descriptions presented here are taken from the interview stimuli. They are therefore texts that were presented to the study participants in order to gain an impression of the technology.

Three examples were randomly assigned per study participant to collect sufficient data on each technology example in a staggered and alternating process. The goal of this assignment was to obtain diverse responses from study participants, regardless of participant characteristics – thus allowing each participant the opportunity to respond to each (randomly assigned) device without having to have prior experience.

The following main questions were asked in the interviews (in the event of further clarification, the interview responses were addressed in greater depth with additional questions):

- What do you think about the technology in general?
- What could be the advantages or problems of using the technology at this ward?
- How would you imagine the reaction of patients to the device?
- Would you or would you not use the device and why?
- How do you think the way you are cared for or the way patients are cared for in general changes when using this device?
- Do you think the device could influence nurses and their work and if so, how?

It was expected that 20 interviews would be ideal to ensure sufficient information and allow for the theoretical generalisation of findings (Polit and Beck, 2010). From this number, we expected a nearly equal distribution of sampling criteria (see next subsection). Nonetheless, interim analyses were used to test whether discontinuation was possible at an earlier stage if no new information was added. The outcome of this will be reported at the beginning of the results section. The first interview was conducted as the pre-test, indicating problems in question formulation and arrangement.

### 2.2. Recruitment strategy and interview procedure

A criterion-based, purposeful sampling strategy was adopted for the study (Patton, 2009). Patients are supposed to represent different experiences in health care and can share their expectations and attitudes toward the presented technology. The project ward treats a wide range of patients of different age groups and with many different conditions (which is why it was selected as appropriate for the research project). For this reason, the sample for the study should reflect the experience of patients on the ward who could also be potential users of new technology at a later post-implementation period. A group also relevant to this question consists of patients with dementia, but they could not be interviewed for the study because corresponding ethical and methodological challenges could not be addressed as part of the study.

Sampling criteria to approach such study participants were: (1) being a patient on the hospital ward of the research project, (2) gender, (3) age, and (4) the reason for hospital admission [elective (by appointment) or by emergency]. The first and fourth criteria should be fulfilled, so that the distribution of the patients should correspond as closely as possible to the implementation conditions of the project station (trauma surgery with a significant distribution of elective surgeries). The criteria gender and age refer to the different social situatedness of potential users of technology: Regarding gender, for example, UNESCO already stated in 2007 [besides decades of debates in feminist science and technology studies (Haraway, 1988)] that women should be systematically involved in the design and evaluation of new technologies as the gendered perspective on technology can differ significantly (UNESCO, 2007). The same applies to the perspective of different age groups, which should be taken into account in the design and evaluation of technology, especially when technology in the context of care is often directed at older persons (Nierling and Domínguez-Rué, 2016). The criteria were expected to be equally distributed for recruited study participants, except for the first criterion (all participants were sought to be from the ward). In addition, all patients needed to be able to give informed consent.

According to the principles mentioned above, participants should be patients in the associated ward at the time of the interview. To find suitable and participating patients, four professional nurses from the ward were assigned to identify participants and asked for their general willingness and interest to participate in the study. These nurses were previously introduced to the selection criteria and were able to ask the research team at any time if they had uncertainties about a patient’s fit. This procedure made it possible to carry out the recruitment even without the permanent presence of members of the research project, as they could not be constantly on-site during the ongoing practical operation of the station. Those patients who were interested were informed by a researcher on more study details and given information on study principles like electronic voice recording, data security measures and ethical considerations. If patients consented to participate in the interview, they were asked to sign the consent form. No financial incentives were given. Participation was voluntary.

The interview started with the activation of a recording device. After the technology stimulus was given (a short introductory text, that was written by the interviewer, was read out while a video about the technology was shown to the patient), the questions from the semi-structured interview guideline were asked. Afterwards, patients filled out a short questionnaire regarding socio-demographic details. Shortly after the interview, the interviewer made notes on essential data (e.g., duration, atmosphere) and reflections on the procedure. It was assumed that the interviews would be 30–45 min long.

### 2.3. Data analyses

The audio files were transcribed word-by-word and analysed using the evaluative qualitative content analysis (Kuckartz, 2018). In this methodological version of content analysis, general evaluative categories are formed (with a positive and negative manifestation), which are derived in a deductive-inductive mix: This means that main categories are developed with regard to the research question and reflect the state of the research literature – these are the deductive categories (Green and Thorogood, 2004a; Kuckartz, 2018). These categories are also considered the starting point for coding the data. During data coding, if new topics emerge that were not previously considered as main categories, these will be included as new categories in the analysis – the inductive codes (*ibid.*). This methodological requirement was applied as follows:

*Analysing stage 1*: The first stage contained the development of a category system of main- and sub-codes as described above. Deductive main categories (that derived from the guidelines questions), as well as inductive main categories (that derived from the interview data), were structured using four sub-codes for each main category: (1) a positive and (2) a negative manifestation was used, so that interviewees’ statements can be categorised as generally positive or negative (*ibid.*). (3) Another sub-code was used for ambivalent statements in case participants’ answers were both positive and negative. (4) A last sub-code was used for statements that are not evaluative. More sub-codes were inductively included if interview statements indicated a wider variety of answers. In this mixed approach, a category system could be developed, which created access to the interview material. This was the first step; however, this code system was strongly oriented toward the individual technologies in the interview. Further action was required to evaluate the data *across* the individual technologies to answer the more generally oriented research question.

*Analysing stage 2:* The structured material was reinvestigated in a second coding stage. For this step, all main categories’ positive and negative manifestations were analysed regarding similarities and differences to reduce redundancy. Afterwards, the material was sorted using three perspectives derived from interview data: technology’s expected impact on patients, professional nurses, and the nursing care process. For the transcription and analysis of data, MaxQDA2020 and 2022 were used.

### 2.4. Ethical considerations

Special attention had to be paid to the patient’s well-being so that the interview procedure would not hinder the healing process after surgical intervention. After discussing each technology example, study participants were asked if they needed a break and if they would like to discuss the remaining one or two examples.

The interview setting was the hospital ward, but the specific place of interviewing depended on the patient’s mobility. Mobile patients were taken to a quiet location in the ward. However, since most of the participants were immobile, the interview had to occur in the patient’s room, where interruption occurred occasionally (see limitation). The study received a positive ethics vote from the Hannover Medical School Ethics Committee (issued: 6th of July, 2018, ID: 7933_Bo_K_2018).

### 2.5. Quality assurance

For quality assurance, the COREQ checklist (Tong et al., 2007) was used. All authors planned the study, and all results were discussed with the study team. Author RK (master’s degree) was responsible for the study execution (data collection, transcription, coding, and analysis). He has 10 years of training and 4 years of experience applying qualitative methods as a PhD candidate and research fellow.

The coding and analysis of the data were continuously discussed with scientific peers (e.g., discussions within the study team and scientific colloquiums). Interpretation of interview statements and analytical conclusions were critically reviewed to ensure the reliability of interpretation (Green and Thorogood, 2004a). As a further measure to control the interviewer’s subjectivity, protocol data were incorporated into the analytical process to make the relationship between the interviewer and interviewee transparent. Interview participants could not be contacted to check and validate results because no contact information was taken. Consequently, no repeating interviews were conducted.

## 3. Results

In total, 17 patients were recruited as study participants. The first interview was intended as a pre-test. However, since no significant changes were necessary to the interview guideline, this interview was included. The study did not use a theoretical sampling strategy; therefore, theoretical saturation was not systematically assessed. However, the initially planned 20 interviews were not archived because a lack of new information was observed for the last three interviews – this was considered through preliminary analyses based on the research notes. Hence benefits for the missing interviews were expected to be low.

No interview had to be cancelled. No study participant exercised the right to withdraw from the interview. Because instructed nurses made the initial identification of study participants, no information can be given about the decline of interview requests. All interviews were held in German. Parts of the transcripts were translated for this article by the authors (indicated by [square brackets]).

On average, the total interview duration was 39.6 min (minimum 23 min, maximum 62 min). For an overview of the study participants’ characteristics and technology examples’ contributions for each patient, see Table 2.

**Table 2:** Characteristics of study participants and presented technology per patient.

Category	Manifestation	
*Age*	Medium: 54,5 years / Minimum: 23 years / Maximum: 87 years	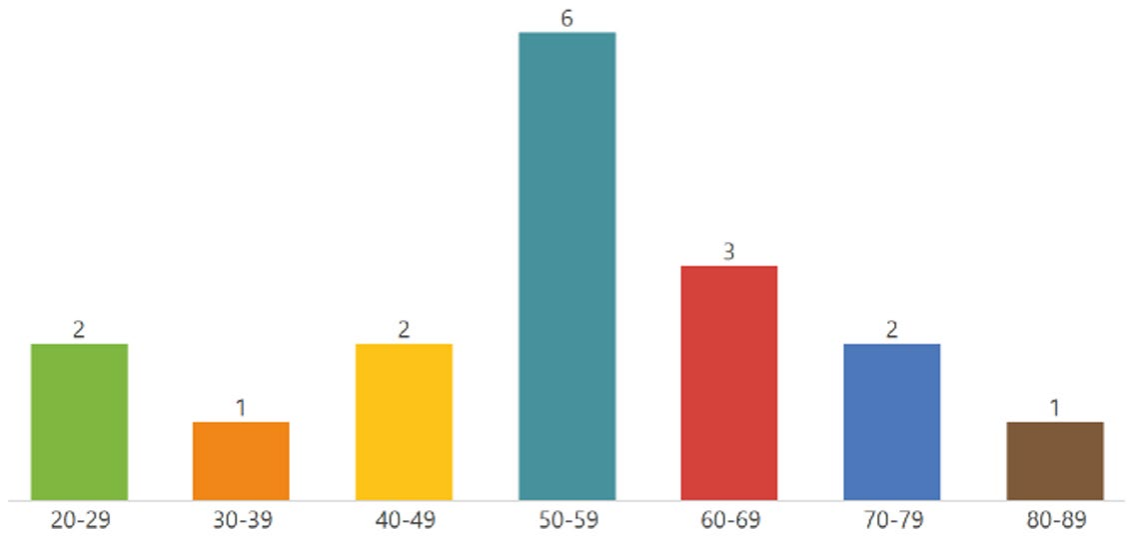
*Gender*	Male: 10 / Female: 7 / Diverse: 0	
*Reason for admission*	Elective admission (planed surgery): 9 / Emergency admission: 8	
*Education (highest degree obtained)*	Lower secondary education: 6 / Middle school: 7 / High school: 3 / University or Collage: 1	
*(Former) Employee status*	Employee (white collar): 8 / Employee (blue collar): 6 / Self-employed: 2 / Others: 1	
*Daily usage of technology*	Smartphone: 13 / PC: 10 / Internet: 14	
*Care level (based on German classification, max. level 5)*	None: 16 / level 3: 1	

	Patient ID	Tec 1	Tec 2	Tec 3	Tec 4	Tec 5	Tec 6	Tec 7	Tec 8
Blue fields indicate presented technology to patient*	P_T								
	P001								
	P002								
	P003								
	P004								
	P005								
	P006								
	P007								
	P008								
	P009								
	P010								
	P011								
	P012								
	P013								
	P014								
	P015								
	P016								
		7	7	7	6	6	6	6	6

*The staggered and alternating rotation of technologies from participant to participant was realised, but deviations from the selection scheme occurred in individual cases. In these cases, hints emerged before the interview that certain participants would likely provide interesting feedback on specific technologies. In this case, the study investigators spontaneously decided to present technologies other than the pre-selected ones – the not discussed examples were then presented in subsequent interviews so that all technologies could be discussed with approximately equal frequency.

**Figure 1:**
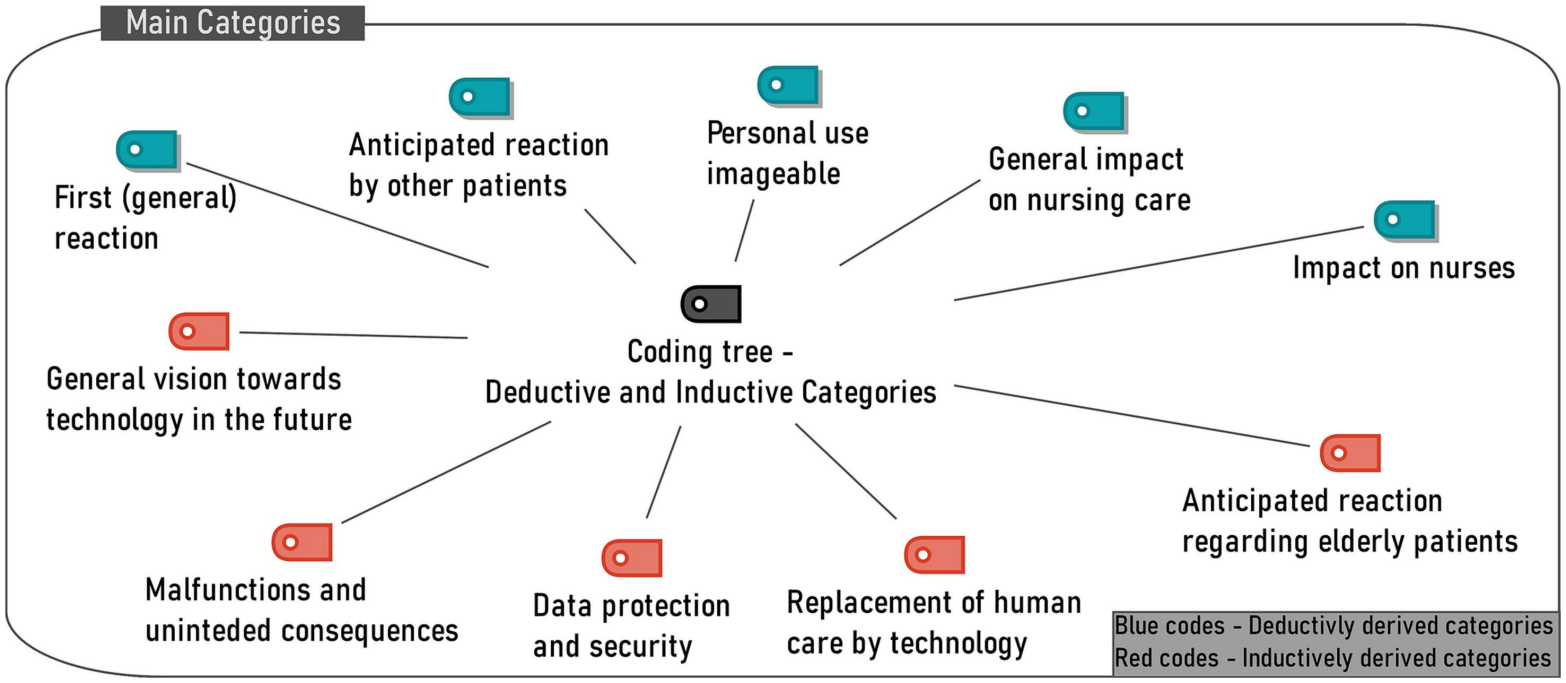
Coding tree of main categories (Analytical stage 1).

In the first analysing stage, a code tree was developed from the interview material in a mixed inductive-deductive procedure – for an overview of the coding tree, see Figure 1.

Since it was not part of this study to have the presented technologies assessed individually, the overall evaluation of the technologies for each main code is not addressed here. However, a quantitative summary of information on positive or negative manifestations of interview statements per technology and main code can be found in the Appendix.

**Figure 2:**
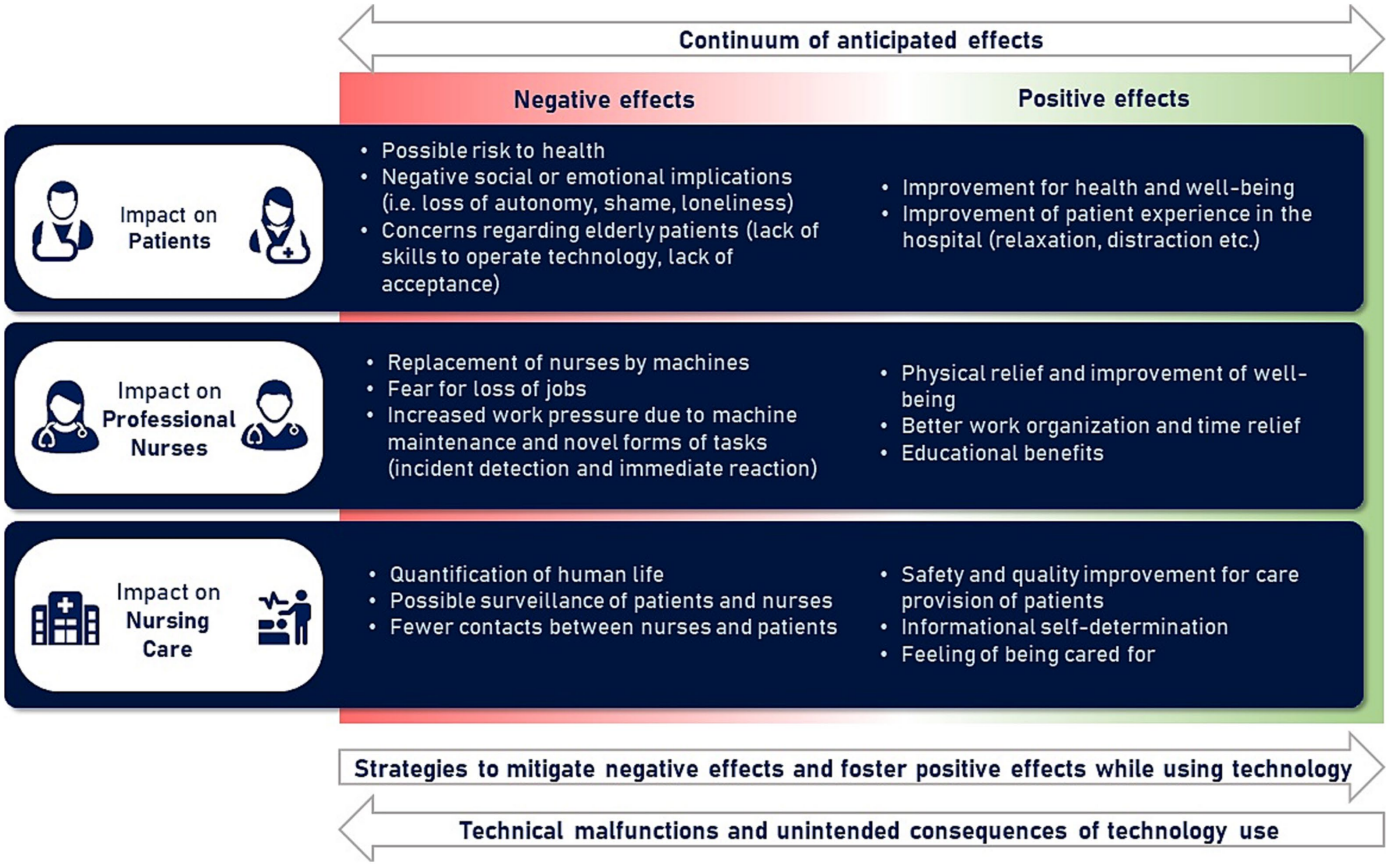
Continuum of anticipated effects (Analytical stage 2).

The result of the second analysing stage can be described as a continuum of anticipated effects for implementing nursing technology, as seen in Figure 2. This continuum will be presented in the following section by introducing each aspect of the scheme, starting from the anticipated impact of technology (1) on patients, (2) on nurses and (3) on the nursing care process while comparing potentially positive and negative outcomes. In another section (4), strategies and ideas of patients to foster positive technology effects and avoid unintended consequences are reported.

### 3.1. Perspective 1: impact on patients

#### 3.1.1. Positive effects

For each of the three perspectives in the schema, the introduction of nursing technology can lead to negative and positive consequences or effects. Positive effects for patients, in general, are seen in **improved health and well-being** and **better patient experience** in the hospital. All presented technologies are expected to affect patients’ health, healing or well-being positively – except the nursing glasses, where no such expectations could have been found and that were designed for documentation and educational reasons. For example, when a participant was asked whether she believes that the re-positioning system for the prevention of pressure ulcers might be beneficial for patients, she replied:

'[Well, of course, simply because you can sleep throughout the night and no nurse needs to wake you up in the night]' (P005, para. 76).

Another participant was asked the same question regarding the cat robot:

'[Whether the system may help? Of course. Because it gives people comfort, relaxation and calmness]' (P012, para. 107).

Introduced technology may also improve the hospital stay and experience for patients. For example, one participant responded to the multimedia bedside monitor that is technically capable of room control:

'[Well, if you say it can control windows and the temperature, that would be a nice thing if you cannot leave the bed, like me. I have to wait all the time […]]' (P016, para. 88).

The bedside monitor may increase autonomy in the care of patient immobility.

In another case, a patient was asked whether he would use the hospital transport robot, and he responded:

‘[I would. […] Instead of waiting for my coffee for an hour, I would use it […]. I understand that patients are lying here waiting for the nurses because they have something else on their minds. What is perhaps even more important than the one who is now upset here that he does not get his coffee]' (P012, para. 81).

The patient described a situation in which he acknowledged that patients have different needs. The more urgent case requires more attention from nurses. The patient would step back if a machine could fulfil his wish for a hot beverage.

#### 3.1.2. Negative effects

In contrast, patients also described the **potential for adverse effects**. Although they were not often expressing concerns about unintended harmful consequences of technology (compared to potentially positive impact), some problems can be found about each of the presented products. For instance, one patient expressed his concerns regarding the hand exoskeleton:

'[The more you get supported, the more your muscle function decreases. Of course, I would be afraid of that]' (P004, para. 86 to 88).

Regarding the automatic positioning mattress system, one of the participants wondered:

'[And the security is given? [Interviewer: Security for what?] That you do not fall out. That would be the biggest factor for me. Everything else is good]' (P008, para. 85 to 87).

Although the patient acknowledged the device’s health benefit, he saw a real risk to the patient’s health if security was not given.

Technology may also have significant **negative emotional or social implications**. For the robot cat, one patient remarked:

'[Well, I know the situation in […] nursing homes, and I see that the people there are also lonely. And how scarce the staff is there. Therefore I have the fear immediately, one gives them the cat and then […] staff is saved]' (P014, para. 104 to 142).

This study participant was worried that the robot contributes to patient loneliness. Other concerns regard the positioning system (patients may lose their autonomy) and the incontinence detection mat (the sensor will make a person’s bedwetting visible and trigger shame).

Some study participants saw **negative consequences for older patients** confronted with new technology. They anticipate a lack of skill by geriatric patients to familiarise themselves with and operate the technology or a lack of will to use the device. For instance, one interviewee commented on the bedside monitor:

'[I do not think you can cope with it when you are older. They […] might just manage to get the bed up and down and so on, but barely manage to get heating on, the light off]' (P016, para. 94).

One patient responded similarly to the intelligent walker by saying:

'[I could imagine that with older people, […] they would say “Nope. I cannot cope with that”]' (P012, para. 59).

Most study participants believed that with a careful introduction, this group could also be introduced and supported with technology (see the section on mitigation strategies).

Participants of a relatively younger generations (<49 years of age) tend to have a more positive, optimistic attitude toward the presented technologies and vice versa. Ambivalent or critical attitudes about the presented technologies are more common from the middle and high educational levels. For example, participants with a middle or higher educational level tend to imagine that there might be more technology malfunctions. However, all age groups tend to imagine that they would use the devices themselves and that they could positively influence patient care (see perspective 3).

### 3.2. Perspective 2: impact on professional nurses

#### 3.2.1. Positive effects

For professional nurses, there are several positive effects anticipated. For six of eight technologies, the technologies are expected to **support nurses** in reducing physical stress (mobilisation of heavy patients, relieving walking routes), enable prioritisation of tasks, and release nurses from time-consuming tasks like documentation or providing support in times of staff shortages.

Regarding the bedside monitor, one patient commented on the system function to send patient wishes to nurses:

‘[That would be better for the nurses. If you had something like [this system], if you just want something to drink. They then know that. Well, if others have to go to the toilet quickly or something, […] then it takes a little bit of time]' (P002, para. 52).

Another patient talked about the potential effect of the transport robot:

‘[As support for reducing the walking distance to make the workday more manageable, perhaps to bring blood samples to the laboratory, insofar as that is possible at all over the floors]’ (P007, para. 64).

The use of the transport robot can lead to a reduction in walking distances. However, the infrastructural requirement in the building must be given.

The only device where patients see an advantage in **training professional nurses** is the nursing glasses:

‘[Yes, that is great for training. Of course, it is really great because the trainees are then shown that you have to do this and that]’ (P_T, para. 72).

#### 3.2.2. Negative effects

Patients also see the potential negative impact of technology on nurses. For six of the eight presented technologies (except the hand exoskeleton and the incontinence detection mat), interview passages could be identified in which patients suggest that the device could **replace human labour and reduce the number of jobs**. A participant responded to the intelligent walker:

‘[I see a problem in the fact that jobs are lost as a result]’ (P012, para. 57).

This feared reduction in the workforce is linked to a possible decrease in the quality of health care (see next section). Although men and women generally discussed similar topics about the technologies presented, the assessment of women toward the technologies was more critical than for men, especially concerning the overall evaluation of the technology, the possible additional burden of new technology for nurses or the worsening of the care relationship (see perspective 3). Older participants (>60 years of age) are also more likely to consider the replacement of caregivers with technology than other age groups (<49 and 50–59 years of age), where such concerns are less frequently expressed. However, many of these passages also show that caring and emphatic motives prevail for such concerns. The study participants in general do not want professional nurses to be replaced by machines and thus lose their jobs.

Some technologies are expected to imply an **additional burden on nurses**. For instance, a patient feared that the robotic cat might provoke situations where more patients want robots than available. The patient also wondered whether this could lead to emotionally stressful situations for the patients, for example, when they have to hand over the robot when discharged from the hospital. A patient noted that an alert to the bed-exit function of the incidence detection mat requires an immediate response:

‘[If there is an immediate reaction, that is okay. That is very important because one must react immediately. […] It must be because, if they leave the bed is already dangerous, isn't it?]’ (P002, para. 66).

An immediate reaction should be necessary for the nurse. Therefore, reaction time for nurses shortens.

### 3.3. Perspective 3: impact on the nursing process

#### 3.3.1. Positive effects

The third perspective described by patients is that of nursing care and encompasses the views of patients and nurses in their interaction. Patients describe ways to **improve nursing care** for all presented technology examples. For instance, for the incontinence and bed-exit detection mat, a patient mentioned:

'[That is positive surveillance. Because you are not always so close that you notice what is happening, [when using the technology] you are warned]' (P010, para. 60).

Another aspect is due to **informational self-determination** and free access to information regarding personal health information. For instance, for the bedside monitor that can display personal medical data, a patient said:

'[And what is also very good: looking at your treatment plans, […] so that one has a bit more information]’ (P_T, para. 36).

Other patients described that in a few situations, they would also like to decide that personal data should not be shared, and they would also like to move freely and anonymously in the hospital (for example, if they want to eat something unhealthy or smoke a cigarette). Hence, a self-determined choice of information to share is essential for these individuals.

#### 3.3.2. Negative effects

In addition to the positive impact that patients see on nursing care, they also anticipated adverse effects. One of these effects is the concern about the **dehumanisation of people in need of care**, as described in the case of the transport robot:

‘[I would reject it. […] I think you are already enough of a number as a human being. […] You are happy as a human being, as a patient, if there is the social component and the human emotional reaction of a doctor]' (P007, para. 72).

In contrast to the assumption above, it is assumed that the technology does not contribute to the increased availability of time resources. Technology is feared to cause a state in which people are increasingly understood as numbers. Instead of a person in need, they may become an object to be treated.

Some patients are concerned about the **contact with nurses, which could be significantly reduced** in the case of the bedside system:

‘[So I think it is terrible. I mean, technology is already more or less everywhere […]. Not even ordering my food personally? […] I do not have any contact with the nurses hardly at all? Terrible. No thanks]' (P008, para. 103).

While merely a few passages were identified in the interviews in which patients were concerned about the **constant surveillance** that could result from new, digital technology, there were references to such concerns (especially for the incontinence and bed-exit detection mat).

### 3.4. When things change: mitigation strategies, malfunctions and unintended consequences

Positive and negative anticipated effects are often opposite consequences of similar origin. For example, a device that improves the health and well-being of patients becomes a health risk the moment a malfunction occurs. Similarly, a device that opens time resources and physical relief for professional nurses becomes a burden to patients and nurses in case of replacing humans with machines.

Patients reflect on the interrelatedness of nursing technology’s positive and negative impact and identify strategies to foster positive effects (see Figure 2, arrow at the bottom to the right). Such strategies could be identified for various problem areas (especially on data protection and the involvement of older patients). For example, one suspected that the hand exoskeleton might not interest older people needing care. However, he also added:

'[I think that some would perhaps be more open if you explain what [the exoskeleton] are there for]' (P002, 86).

In opposition to these strategies, study participants were concerned about technical malfunction and other negative unintended consequences (see Figure 2, arrow at the bottom to the left). Electively admitted participants (i.e., those who came to the hospital via a scheduled procedure) tend to see more potential threats to data privacy from the technologies and are less often talking about possible malfunctions of the devices compared to patients admitted on an emergency basis. Such consequences can both be shown using the example of the intelligent rollator:

'[I don't know how advanced that is, whether that thing may roll by itself when I go and the technique is broken and the rolls go by itself]' (P001, para. 82).

In another interview regarding the same technology, a person said:

'[In the hospital, it is certainly a support, right? And if I am not allowed to take it home? […] People are accustomed to such things. And if they are no longer available, then it could be that many fall back into an old routine]' (P012, para. 52).

## 4. Discussion

The study shows that patients identify a spectrum of possible effects of technology implementation and critically weigh up opportunities and challenges. It is often the same feature of technology that can have a positive and negative impact. For example, some patients believe that the robot cat may have a calming effect on dementia patients. At the same time, its use could simultaneously reduce the contact time with nurses and could increase the individual’s social isolation. Similarly, the effects of technologies are usually relevant at the same time from different perspectives (patients, nurses, nursing process) since they are intertwined. For example, the fall detection mat is expected to provide better patient safety. However, it is feared to add additional workload on nurses due to the need to carry and operate a smartphone and a required immediate response in the event of fall detection.

Thus, the spectrum in this study represents not only the possible effects and outcomes of technology. It represents patients’ positive and negative expectations, which reflect emotional reactions to the outcome of technology use and equates positive implementation outcomes with health-promoting effects and the ones that are negative and may in turn jeopardise health and well-being. Thus, the patient’s responses illustrate that the use of new technology can have a direct impact on the emotional and physiological state of its users.

From the patients’ point of view, the eight technologies presented cannot be assigned to a fixed position on the derived continuum since technology is not seen to have a fixed positive or negative impact. Moreover, implementation conditions and application conditions determine whether a technical product tends to produce a positive or negative influence. It was shown elsewhere, using the example of a nursing documentation system, that learning effects and adaptation effects to new technology can also have an influence on subjective usefulness by the users, which is why the assessment of an overall positive and negative impact of technology varies over time (Zadvinskis et al., 2018).

As has been shown in other studies [for the case of professional caregivers see (Kaihlanen et al., 2022)], our study confirmed that patients do not have a static but a relational understanding of technologies [see also (Friesacher, 2010; Beedholm et al., 2015)]. Application contexts, implementation conditions, and decisions about how new technology is used in practice play a central role for patients in our study. Three implications for the adoption and use of care technologies can be drawn from this.

### 4.1. Implication 1: the fundamentals of care as a reference point for sound implementation of nursing technology

The relational understanding of technology, as described by the patents in our study, represents the openness of how technology is used in practice, which principles guide action, and accordingly, which goal and which (positive or negative) effects are achieved. The example of Crocker and Timmons show how nurses’ use of a weaning device changes a ‘medical technology’ to a ‘nursing technology’ while using it in a logic of nursing care (Crocker and Timmons, 2009).

This logic of care can be illustrated with the concept of ‘fundamental of care.’ This concept focuses on the relationship between caretaker and caregiver and integrates physical, social and psychological aspects within the care delivery instead of merely depersonalised care of the body (Kitson et al., 2014). Looking now at the core areas of nursing care, physical, emotional, cognitive, and organisational work (Jackson et al., 2021), these areas can be found on our continuum of anticipated effects. In the case of technology supporting the fundamentals of nursing care, it is also expected to be beneficial. Conversely, when the fundamentals of care are questioned by the way technology is used and when human nursing logic is to be replaced by a ‘mechanistic and technical provision of care’ (Stayt et al., 2015; Archibald and Barnard, 2018), its use is viewed critically or rejected.

### 4.2. Implication 2: patients’ concerns about negative implementation consequences for caregivers demonstrates a form of mutual care

At least in the German context (which is also the context of our study), digital technologies are only slowly finding their way into healthcare, and stakeholders (e.g., physicians or patients) have so far tended to use technology for healthcare applications independently of one another with little interaction (Albrecht et al., 2017).

Our interview study, however, shows that patients take the perspective of nurses to think about how new technology might impact them and their work. The concerns they express regarding new technology may depend strongly upon the context of inpatient care, where patients are dependent on round-the-clock care from nurses and are thus also affected by adverse effects on them [this had been described as a notion of a ‘mutual vulnerability’ by Angel and Vatne (2017)].

The perspective of nurses that many patients took in the interviews is separate from an accurate anticipation of how nurses would perform it themselves. Indeed, genuine nurses’ perspectives are not the subject of our study. Nevertheless, patients’ concerns about a possible decline in the care relationship are relevant, as they show which version of the future is desirable from the patient’s point of view and which should be rejected. Thus, patients’ concerns about job loss for caregivers and the replacement of human caregiving by robots can be described as a notion of mutual care that should not be changed even with the increasing use of technology. The second implication is that if the situation of nurses changes, the situation of patients also changes. That is why patient anticipation matters, because of their usually vulnerable position in health care, from which negative trends affect them directly or indirectly.

### 4.3. Implication 3: the role of patients in technology implementation does not change but may be influenced by technology implementation

Patients have multiple roles that can be described as ‘patient as person’ and ‘person as patient’ (Fassin, 2008). As a ‘person as patient,’ they must take action on their treatment success and endure pain and other accompanying symptoms while maintaining control over their body. In contrast, as ‘patient as person,’ they must negotiate through the health care situation and maintain their sovereignty and privacy (*ibid.*). Looking now at the area relevant to patients during the introduction of new technologies (see Figure 2), the role of ‘technology user’ is not a new third role for patients in the healthcare institution. Instead, the effects initiated by technology (new responsibilities, potential benefits, problems) are not to be considered in isolation but interwoven with aspects of the patient experience already taking place in the care setting.

Here, technologies offer an opportunity, if used consciously and purposefully as part of a comprehensive health intervention, to have an impact on the experience of illness and the perception of one’s own body – using the example of breast cancer survivors, it was demonstrated how a targeted intervention could positively influence the perception of the body and mental health, see (Sebri et al., 2022). Similar to the expectancy of patients in this study, a systematic review found that robots, that are used in the health care system, may lead to multiple positive effects for physical (medication adherence), mental (mental mood, cognitive capabilities), and social health promotion (companionship, facilitation of social connections) (Huang et al., 2023). Hence, technology in this context can be viewed as complementary to aspects of the patient experience but does not lead to a fundamental reassembling of the areas that determine a positive or negative patient experience. In this context, even a ‘good’ technology cannot exclusively determine a ‘good’ patient experience. However, it can support a resonant, positive relationship in the care setting between nurses and patients [for a theoretical account of the theory of resonance, see Rosa, 2019].

### 4.4. Strengths and limitations of the study

The strength of this study is the openness with which the patients were able to respond to the presented technologies. Apart from general questions about advantages and disadvantages and application scenarios, no thematically specific questions were asked. The study participants could freely discuss topics in the interviews that were relevant to them.

The following points represent limitations of the study:

*First*, the recruitment strategy. Since the study team had no permanent access to the ward, the recruitment process had to be initialised with the help of nurses. It cannot be ruled out that other than the recruitment criteria were the deciding factor in the selection of study participants, such as sympathy or antipathy toward potential participants.

*Second*, many participants were not mobile. Therefore, the mobilisation of patients for the course of a quiet interview place would have been potentially stressful for study participants and consequently collided with the principle of the interview not interfering with patients’ healing process. Therefore, in some cases, interviews needed to be conducted in the patients’ room with health care professionals, other patients and visitors interrupting the interview. However, all interviews were finalised, and the study team has no information about any inconveniences to patients’ healthcare situation due to participating in the study.

*Third*, as described at the beginning of the methods section (2.1, *study design*), study participants were presented with video clips and text descriptions as interview stimuli, which they were asked to use to respond to the technologies. This could have meant that the patients could not form their own opinion of the devices and were influenced by the chosen form of presentation. Therefore, it is possible that the patients would have been given different answers if they had the devices in front of them. However, it was impossible for the study team to have all eight technologies on-site, as the financial project resources were insufficient for this, and patient care would have been significantly disrupted.

## 5. Conclusion

The anticipation work done by the study participants is separate from an accurate prediction of effects but rather an insight into social and psychological complexities that influence future trends (Saritas, 2013). In a targeted intervention, not only conscious, intended effects and consequences of actions can be found. Also, there are effects that none of the interveners intended (unintended consequences) or predicted (unanticipated consequences) (de Zwart, 2015; van Manen, 2015). These consequences illustrate why the contribution of various stakeholders – in this case, hospital patients – in designing and enforcing planned public action is relevant: Those who add their reflections on possible implementation effects draw on their knowledge, experience, and social values, which may differ between these groups (Weiss et al., 2018).

The central finding is that patients do not envision new technology as a fundamentally positive or negative development for nursing care. The study participants critically assessed opportunities and challenges and formulated strategies to foster positive or mitigate adverse effects. With a few exceptions, most patients favoured the presented technologies in certain circumstances. Nevertheless, they see potential threats to care and safety in healthcare if technologies are used not in a supportive manner but in such a way that human care is replaced and care is provided mechanistically.

The developed scheme of a continuum of anticipated effects can provide a point of orientation to support future technology introduction processes by anticipating the possible positive and negative impact of implementation and utilisation processes. The main benefits would be advantages for the health, well-being, and experience of patients in the hospital, as well as improvements in the quality of health care, which would be accompanied by improvements in working conditions for nursing staff. There are fears of possible negative effects on health and well-being and fears of far-reaching dehumanisation of care caused by the replacement of human professionals with machines. In addition, the scheme can help to avoid technology-deterministic and -centred strategies by considering technology integration into the human-centred care process that also involves the perspective of patients (Fassin, 2008). Further research may help to take up the schema and incorporate the perspectives of nurses and other stakeholder groups to develop a more comprehensive anticipation tool.

*Clinical implications and further research demand*

1. In the context of nursing, patients will be both (passive) beneficiaries and (active) users. The design of technologies and the strategies to bring them into practice must take into account different abilities, prerequisites and expectations of patients with different backgrounds. This study did not investigate the perspective of patients with dementia, who represent a large group with specific needs and should be considered in further research activities.
2. Patients’ competencies in the use of technology should not be taken for granted and should be strengthened in a targeted manner. Further research should be conducted together with stakeholders on how the individualised use of technology can be designed for patients in different care settings.
3. Patients have expectations and fears about health-related interventions in the hospital, and this is especially true for novel technology with which there is little experience (as in the example of autonomous robots). As with other interventions in healthcare settings, the use of new technologies and their potential advantages and disadvantages should be highlighted and explained. The development of appropriate informational material and training of nursing staff, not merely in the use of technology but also in communicating it to patients, are crucial.
4. Implementation strategies should actively take into account fears from the beginning, such as those shown in this study, and in particular, promote the use of technology that is assistive and not a substitute for human care. A particular difficulty here is the introduction of systems that can act autonomously. Here, the consent of patients to use this kind of technology should be given, since patients may have concerns about (social) interaction with machines (in situations of care that are often characterised by vulnerability).
5. The support of social interaction between nurses and patients can be a useful outcome to assess the effectiveness of new technologies in nursing care. However, appropriate methodological requirements (i.e., instruments) for this are lacking.

## Data availability statement

The original contributions presented in the study are included in the article/Supplementary material, further inquiries can be directed to the corresponding author.

## Ethics statement

The studies involving humans were approved by Hannover Medical School Ethics Committee. The studies were conducted in accordance with the local legislation and institutional requirements. The participants provided their written informed consent to participate in this study.

## Author contributions

M-LD and RK: conceptualisation, methodology, and validation. RK: data collection and formal analysis. RK, U-VA, and M-LD: investigation, writing—original draft, and writing—review and editing. U-VA and M-LD: supervision and project administration. M-LD: funding acquisition. All authors contributed to the article and approved the submitted version.

## Funding

This study was funded by the German Federal Ministry of Education and Research, funding number 16SV7892K. This publication is funded by the German Research Foundation (DFG) under the program: “Open Access Publication Costs”.

## Conflict of interest

The authors declare that the research was conducted in the absence of any commercial or financial relationships that could be construed as a potential conflict of interest.

## Publisher’s note

All claims expressed in this article are solely those of the authors and do not necessarily represent those of their affiliated organizations, or those of the publisher, the editors and the reviewers. Any product that may be evaluated in this article, or claim that may be made by its manufacturer, is not guaranteed or endorsed by the publisher.
