# Supplementary material for: Expectations of new technologies in nursing care among hospital patients in Germany – an interview study
Source: Front Psychol. 2023 Sep 14;14:1227852. doi: 10.3389/fpsyg.2023.1227852 (PMC10539617; doi:10.3389/fpsyg.2023.1227852)
Supplement: Supplementary file 1 [file Table_1.DOCX]

Supplementary Material

Expectations of new technologies in nursing care among hospital patients in Germany – An interview study

Ronny Klawunn^*^, Urs-Vito Albrecht, Marie-Luise Dierks

*** Correspondence:** Corresponding Author: Ronny Klawunn, Klawunn.ronny@mh-hannover.de

# Supplementary Figures and Table

## Supplementary Table 1: Quantitative summary of evaluative codings by category and technology (analysing stage 1)

|  | 1. Bedside Monitor | | 2. Nursing glasses | | 3. Positioning system | | 4. Robot cat | | 5. Intelligent Walker | | 6. Transport robot | | 7. Hand exoskeleton | | 8. In-continence detection | | Sum | |
| --- | --- | --- | --- | --- | --- | --- | --- | --- | --- | --- | --- | --- | --- | --- | --- | --- | --- | --- |
| No. of presentations in interviews | 7 | | 7 | | 7 | | 6 | | 6 | | 6 | | 6 | | 6 | | 51 | |
| First (general) reaction – positive/negative | 3 | 1 | 7 | 0 | 7 | 0 | 4 | 3 | 6 | 0 | 4 | 1 | 6 | 0 | 6 | 0 | 43 | 5 |
| Anticipated reaction  by other patients – positive/negative | 0 | 0 | 5 | 0 | 3 | 3 | 4 | 3 | 1 | 1 | 0 | 1 | 2 | 1 | 1 | 0 | 16 | 9 |
| Personal use imaginable – yes/no | 5 | 1 | 1 | 2 | 6 | 0 | 0 | 0 | 3 | 1 | 5 | 0 | 5 | 1 | 4 | 2 | 29 | 7 |
| General impact on nursing care – positive/negative | 6 | 2 | 4 | 0 | 3 | 2 | 4 | 1 | 5 | 1 | 6 | 2 | 3 | 1 | 4 | 0 | 35 | 9 |
| Impact on nurses – support/burden | 4 | 0 | 3 | 0 | 3 | 0 | 1 | 1 | 0 | 0 | 2 | 0 | 2 | 0 | 0 | 1 | 15 | 2 |
| Anticipated reaction regarding elderly patients – positive/negative | 2 | 3 | 1 | 0 | 0 | 0 | 0 | 0 | 5 | 1 | 0 | 1 | 2 | 0 | 0 | 0 | 10 | 5 |
| Replacement of human  care by technology – hard to image / imaginable | 1 | 4 | 0 | 1 | 3 | 3 | 1 | 1 | 0 | 1 | 1 | 4 | 0 | 0 | 0 | 0 | 6 | 14 |
| Data protection and security – no issues/problematic | 1 | 2 | 2 | 1 | 0 | 0 | 0 | 0 | 1 | 1 | 1 | 1 | 0 | 0 | 0 | 1 | 5 | 6 |
| Malfunction and unintended consequcens – hard to image/imaginable | 0 | 0 | 1 | 0 | 0 | 2 | 1 | 0 | 0 | 3 | 0 | 0 | 2 | 0 | 0 | 1 | 4 | 6 |
| *Number indicated provided information given by patients to specific technology and category, multiple information given by patients per category and technology are summarized (for example, if a patient anticipated negative effects on nurses by a technology multiple times, its response is counted as 1).*  *Ambivalent interview statements (both positive and negative in one statement) are not mentioned in this table.*  *Inductive Code “General vision towards technology in the future” is not mentioned in this table because answers were not focused on the presented technology.* | | | | | | | | | | | | | | | | |  | |
